# Maize grain yield enhancement in modern hybrids associated with greater stalk lodging resistance at a high planting density: a case study in northeast China

**DOI:** 10.1038/s41598-022-18908-z

**Published:** 2022-08-27

**Authors:** Jingjing Cui, Zhengguo Cui, Yang Lu, Xiaofei Lv, Qingjun Cao, Yunlong Hou, Xiangyu Yang, Yan Gu

**Affiliations:** 1 College of Agronomy, Jilin Agricultural University, Changchun, 130118 People’s Republic of China; 2 Jilin Academy of Agricultural Science, Changchun, 130033 People’s Republic of China; 3 Key Laboratory of Northeast Crop Physiology Ecology and Cultivation, Ministry of Agriculture and Rural Affairs of the People’s Republic of China, Changchun, 130033 People’s Republic of China; 4 Liaoyuan Academy of Agricultural Sciences, Liaoyuan, 136299 People’s Republic of China

**Keywords:** Plant sciences, Plant physiology

## Abstract

Lodging resistance is a critical trait in modern maize breeding. This study aimed to examine maize stalk lodging and its related characteristics in response to increasing planting densities in modern hybrids. A two-year field trial was conducted from 2018 to 2019 with two widely grown commercial hybrids (‘Xy335’ and ‘Fm985’) and three planting density treatments of 4.5 × 10^4^ (low density, LD), 6.5 × 10^4^ (medium density, MD), and 8.5 × 10^4^ plants/ha (high density, HD). New hybrid Fm985 had a significantly higher grain yield and lower lodging rate at HD, while there was no significance at LD and MD. Compared to Fm985, old hybrid Xy335 had a significantly high plant height, ear and gravity height, and culm length (CL) across the three planting densities, while opposite stalk bending strength (SBS), dry weight per unit length (DWPU), cross-sectional area, and the cellulose and lignin content in the basal internode were low. Correlation and path analysis revealed that kernel number per ear and lodging rate directly contributed to maize grain yield, while lodging-related traits of SBS, stem lignin, and DWPU had an indirect effect on maize grain yield, suggesting that modern hybrid maize yield enhancement is associated with greater stalk lodging resistance at a high planting density in northeast China.

## Introduction

Maize (*Zea mays* L.), which originated from Mesoamerica and was introduced to China approximately 500 years ago^1^, has become the first major cereals crop in China since 2012 and plays an important role in ensuring an ever-increasing food supply^1^. The Songliao Plain, located in northeast China (containing Jilin, Heilongjiang, Liaoning, and the eastern part of Inner Mongolia of China), is one of the three world-famous Black soil areas, with arable land of 5.68 × 10^6^ ha and maize yield of 2.61 × 10^7^ Mg in 2019, accounting for nearly a quarter of the total national grain yield and about one-third of the total national commodity grain output in China^2^. Thus, maize plays an important role in feeding the nation and is known as the "ballast stone" delivered by the first domestic “White Paper on Northeast Black Land (2020)” in China^3^.

Maize is a widely grown C4 crop with high photosynthetic activity/efficiency, leading to high grain and biomass yield potential globally^4^. The potential grain yield of maize has increased tremendously in the past several decades in China^1^, as well as in other countries such as the US^5,6^ and South Africa^7^. Numerous studies have demonstrated that an increase in planting density is responsible for the increase in maize production, worldwide^8–12^. Lu et al.^13^ reported that maize genetic gain averaged 78.99 kg^−1^ ha^−1^ year^−1^ from the hybrids released from 1999 to 2018 in China, with 54.41% of the total yield gain attributed to breeding. In the US, grain yield increased sixfold from about 1300 kg ha^−1^ in 1939 to 7800 kg ha^−1^ in 2005, with 50% of the yield increase was attributed to breeding effects^14^. The increase in grain yield in modern commercial maize hybrids is mostly due to an increased tolerance toward high planting populations, which is largely attributed to the genetic improvements in crop productivity and associated traits^10,15,16^.

However, increasing planting density usually associated with a high probability of stalking lodging^17^. Stalk lodging not only causes harvest losses by limiting the yield potential of maize cultivars^12^, as well as raises harvest cost due to difficulties in harvest operations^7^. An earlier survey showed stalk lodging resulted in annual yield loss ranging from 5 to 25% in the USA^18,19^, as well as 14% and 28% yield loss for summer maize under root and stalk lodging, respectively at the 12-leaf stage in China^20^, and resulting financial loss of billions of dollars^21^. In northeast China, a wind-induced stalk lodging at the grain filling stage leaded to a yield loss of 14.75% to 29.68% in 2013^8^. Therefore, it’s pivotal to make a balance between grain yield gain and lodging rate in raising planting density for modern hybrids.

Lodging in maize is a complicated phenomenon that is not only influenced by many external factors^22,23^, such as wind, but is also associated with the stalk strength and related traits^21^. The increase in grain yield in modern hybrids may be associated with the plant architecture, stem morphology, and mechanics related to high planting tolerance in genetic improvements. Sergio^24^ reported that genetic gain in modern hybrids is related to the increased number of grains per ear, enhanced post-silking biomass accumulation, and enhanced biomass allocation to reproductive sinks in Argentina. In West Africa, the average rate of increase in maize grain yield is mainly associated with significant improvements in plant and ear stay-green characteristics in the past 22 years^10^, which has prolonged the grain filling duration time and directly affected maize grain yield. In the US, on-farm maize genetic yield gains of released maize hybrids from 1934 to 2004 averaged 115 kg ha^−1^ year^−1^, and the genetic improvement in grain yield was attributed to the continuing phenotypic improvements^23^, such as smaller tassels, more upright leaves, fewer tillers, and a lower grain protein percentage, which can directly or indirectly influence the grain yield. However, the key traits in reducing the occurrence of lodging in maize genetic improvement remain unclear.

The Songliao Plain is the main production area of spring maize in China. Increasing maize planting density is a major approach to maize production. The average maize grain yield increased from 2.44 × 10^3^ kg ha^−1^ in 1980 to 6.87 × 10^3^ kg ha^−1^ in 2019. However, the improvement of maize hybrid genetics plays a key role in maize yield enhancement. ‘Xy335’ is a widely planted maize hybrid planted at a high-density, and it accounted for 40% of the total maize planting area in Jilin province in 2010^8,25,26^. ‘Fm985’ is a newly released local maize hybrid with a similar number of growing degree days (GDD) and plant architecture characteristics as Xy335. However, it is unclear how lodging resistance and related traits respond to increasing plant density.

Therefore, the objectives of this study were (1) to quantify how maize grain yield and stalk lodging rate respond to the increased planting density; (2) to determine how stalk bending strength and related characteristics changed with increased planting density; and (3) to identify the main effects (direct and indirect) contributing to maize grain yield and lodging resistance in modern maize hybrids.

## Results

### Grain yield, yield components, and lodging rate

**Figure 1:**
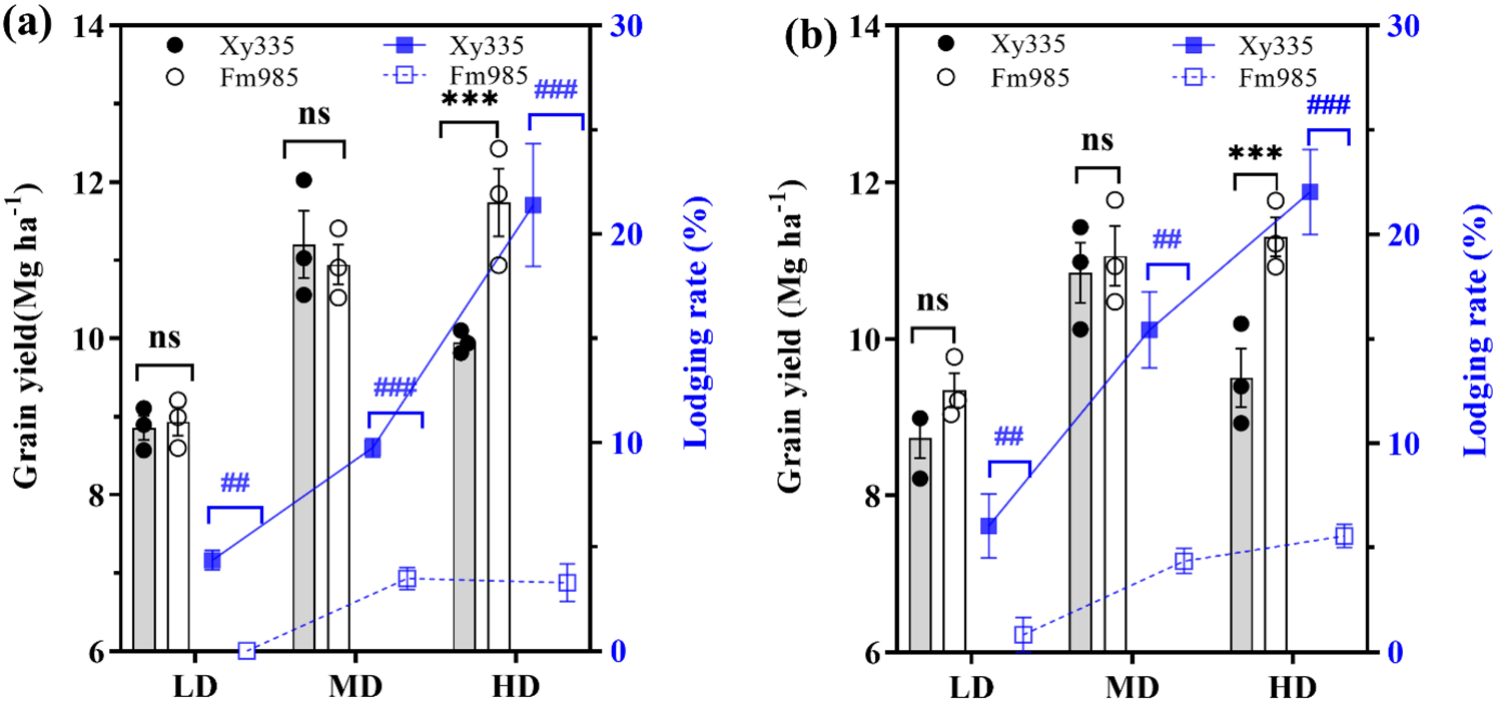
Comparison of maize grain yield, and lodging rate in 2018 (**a**) and 2019 (**b**) at varied planting densities. LD, MD, and HD represent low, medium, and high planting densities, respectively. Bars denote the SE of the mean (n = 3). ns, not significant (*P* > 0.05), ** and *** indicate differences of grain yield significant at 0.01and 0.001levels, # and ### indicate differences of lodging rate significant at 0.01and 0.001levels.

The differences in grain yield and lodging rates between the two cultivars in response to increased planting density were investigated in both years. The results of ANOVA analysis showed that maize hybrid (H), planting density (PD), and their interaction (H × PD) in both years had a significant or highly significant effect on maize grain yield (Fig. 1, Table 1). The grain yield of maize hybrid Fm985 was 4.76% and 8.10% higher than that of Xy335 in 2018 and 2019, respectively, across the average of the three PDs (Fig. 1). For hybrid Fm985, the maize grain yield increased with the increase in PD, while the highest maize yield was recorded at MD for Xy335, which indicated that newly released cultivar Fm985 had a high yield potential at HD.

**Figure 2:**
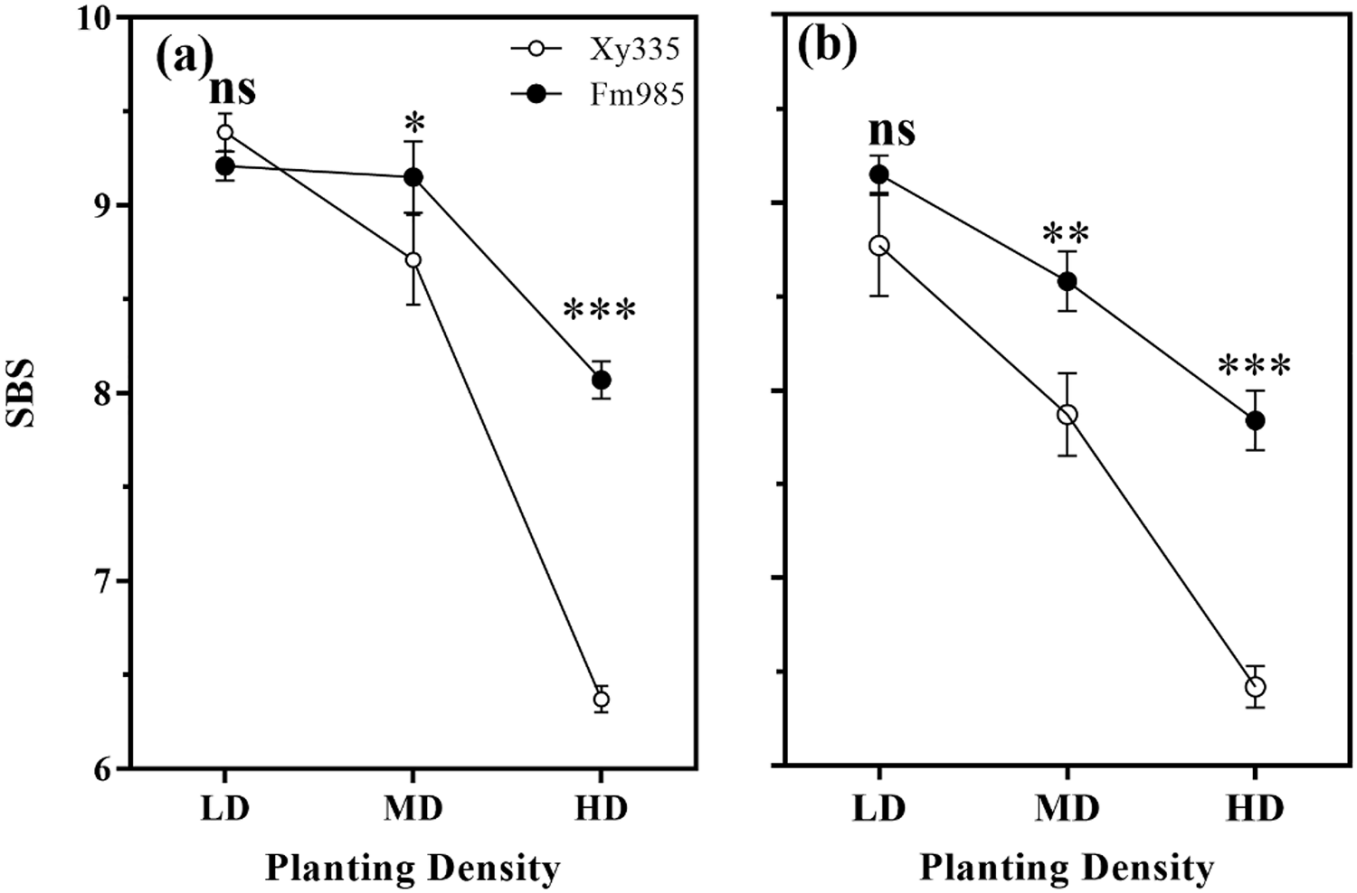
Stalk bending strength (SBS) under different treatments in 2018 (**a**) and 2019 (**b**); SBS were estimated by horizontal pulling resistance value; bars denote the SE of the mean (n = 5); *, ** and ***indicate differences significant at 0.05, 0.01 and 0.001 level; ns indicates no significant (*P* > 0.05).

**Table 1:** Output of three-way ANOVA on the effects of the year (Y), Hybrid (H), planting density (PD), and their interactions.

	Y	C	PD	Y × C	Y × PD	C × PD	Y × C × PD
GY	ns	*	**	ns	ns	***	ns
LR	*	***	***	ns	**	ns	ns
ENPS	*	*	**	*	ns	ns	*
KNPE	ns	ns	**	ns	ns	ns	ns
HGW	**	*	**	ns	ns	**	ns
PH	ns	*	ns	ns	ns	**	ns
EH	ns	**	*	ns	ns	*	ns
GH	ns	*	ns	ns	ns	*	ns
DWPU	ns	*	***	ns	ns	ns	ns
CL	ns	ns	*	ns	ns	ns	ns
CSA	ns	ns	*	ns	ns	ns	ns
SBS	ns	**	**	ns	ns	ns	ns
Cellulose	ns	**	*	ns	ns	**	ns
Lignin	ns	*	*	ns	**	**	ns

GY, grain yield; LR, lodging rate; ENPS, ear number per ha; KNPE, kernel number per ear; HGW, 100-grain weight; PH, plant height; EH, ear height; GH, gravity height; DWPU, dry weight per unit length; CL, culm length; CAS, stem cross-section area; SBS, stem bending strength; Cellulose, cellulose content; Lignin, lignin content.

Maize hybrid (H), planting density (PD) and year (Y) significantly affected yield components, i.e., ear number per ha and 100-grain weight, while kernel number per ear was only significantly affected by planting density (PD) (Tables 1, 2). Therefore, the modern commercial hybrid Fm985 had a higher ear number per ha and 100-kernel weight across the three PDs. Significant differences were observed at HD, while differences were non-significant at LD and MD for both hybrids. Kernel number per ear was significantly affected by PD, whereas it was not significantly affected by hybrid or other factors.

**Table 2:** Maize yield components under different treatments in 2018 and 2019. LD, MD, and HD represent low, medium, and high planting densities, respectively. Mean ± S.E (n = 3).

Year	Hybrid	Ear number per ha (× 10^4^)			Kernel number per ear			100-grain weight (g)		
		LD	MD	HD	LD	MD	HD	LD	MD	HD
2018	Xy335	4.48 ± 0.02	6.12 ± 0.06	8.00 ± 0.07	567.03 ± 17.50	522.28 ± 3.79	482.16 ± 4.70	34.55 ± 0.25	32.49 ± 0.34	28.87 ± 0.24
	Fm958	4.47 ± 0.02	6.29 ± 0.06	8.23 ± 0.07	587.31 ± 8.53	522.28 ± 10.16	475.49 ± 8.95	33.38 ± 0.59	32.80 ± 0.31	32.20 ± 0.64
2019	Xy335	4.46 ± 0.02	6.20 ± 0.05	7.88 ± 0.07	568.64 ± 23.52	521.22 ± 10.52	483.23 ± 5.77	31.59 ± 0.30	29.60 ± 0.89	27.31 ± 0.60
	Fm958	4.45 ± 0.05	6.26 ± 0.05	8.25 ± 0.03	562.03 ± 11.15	522.28 ± 6.90	475.49 ± 6.65	31.40 ± 0.33	30.99 ± 0.12	29.19 ± 0.56

Stalk lodging rates were significantly affected by hybrid (H), planting density (PD), year (Y), and the interaction of PD × Y (Fig. 1, Table 1). The lodging rate of hybrid Xy335 was 5.26-fold higher than Fm985 in 2018, and 4.04-fold higher in 2019 across the three PDs. Among the different PDs, the average lodging rates at LD, MD, and HD were 5.21%, 12.60% and 21.71%, respectively, for Xy335, while they were 0.42%, 3.92% and 4.42%, respectively, for Fm985. The results indicated that the maize hybrid Fm985 had a higher stalk lodging resistance in the present study.

### Stalk lodging-related morphological characteristics

Maize plant architecture-related traits, such as plant height (PH), ear height (EH), and gravity height (GH), were investigated, and hybrid maize Xy335 had a higher PH, EH, and GH in all tested years (Table 3). Compared with low density (LD) and medium density (MD), PH, EH, and GH at high density (HD) had significantly higher values for both cultivars, while no significant differences were observed in these plant architecture characteristics for PD and year, except for EH (Tables 1, 3).

**Table 3:** Plant architecture and stem morphological characteristics under different treatments.

Year	Hybrid	PD	Plant architecture			Stem morphological characteristics		
			PH (cm)	EH (cm)	GH (cm)	DWPU (g cm^−1^)	CL (cm)	CSA (cm^2^)
2018	Xy335	LD	319.37 ± 3.0a	107.91 ± 1.1c	104.34 ± 0.77c	0.79 ± 0.06a	285.33 ± 1.76c	565.87 ± 9.36a
		MD	317.93 ± 2.7a	108.56 ± 0.6c	106.59 ± 0.59c	0.53 ± 0.03c	292.67 ± 3.93b	454.16 ± 9.67b
		HD	327.04 ± 1.3	112.80 ± 1.1b	117.10 ± 1.10a	0.43 ± 0.04d	302.33 ± 3.18a	439.81 ± 24.04b
	Fm985	LD	305.64 ± 2.9b	107.79 ± 1.4b	103.20 ± 1.17c	0.73 ± 0.04a	273.33 ± 4.25d	511.66 ± 18.09ab
		MD	295.34 ± 1.5b	108.62 ± 1.3b	104.85 ± 0.45c	0.65 ± 0.02b	283.00 ± 3.78c	460.54 ± 10.75b
		HD	298.55 ± 3.2b	111.26 ± 1.1a	103.51 ± 1.66c	0.59 ± 0.02b	281.00 ± 3.53c	406.46 ± 17.13c
2019	Xy335	LD	314.34 ± 4.6a	109.64 ± 0.8b	110.41 ± 0.95b	0.77 ± 0.02a	290.00 ± 2.99b	529.84 ± 17.09a
		MD	312.78 ± 5.6a	111.39 ± 21.3ab	113.78 ± 2.10b	0.59 ± 0.01b	289.33 ± 3.84b	436.75 ± 25.91b
		HD	320.52 ± 2.7a	123.63 ± 3.80a	116.94 ± 1.88a	0.47 ± 0.01d	300.33 ± 5.45a	370.24 ± 23.85c
	Fm985	LD	305.32 ± 4.1b	105.31 ± 1.66c	110.84 ± 2.54b	0.75 ± 0.11a	286.00 ± 5.29bc	455.13 ± 31.36b
		MD	300.92 ± 3.3b	106.35 ± 2.11c	105.20 ± 1.96b	0.60 ± 0.02b	285.00 ± 3.21c	438.64 ± 10.89c
		HD	296.04 ± 3.3b	108.65 ± 1.57c	106.95 ± 1.61b	0.56 ± 0.01c	287.67 ± 4.70b	406.08 ± 15.24c

PD, planting density; PH, plant height; EH, ear height; GH, gravity height; DWPU, dry weight per unit length; CL, culm length; CAS, stem cross-section area. Mean ± SE (n = 3). Different lowercase letters in the same column indicate a statistically significant difference at 0.05 level.

The basal stalk morphological characteristics related to stem lodging resistance of dry weight per unit (DWPU) were significantly affected by H and PD; At LD and MD, the differences in DWPU under the same PD were not significant for either cultivars, while hybrid Fm985 had a higher DWPU than XY335 at HD in both years. Culm length (CL) and cross-sectional area (CSA) of the stem were both significantly influenced by PD, while no significant effect was observed in H, Y, and other interactions (Tables 1, 3).

### Stalk bending strength (SBS) and carbohydrates content of basal internode

**Figure 3:**
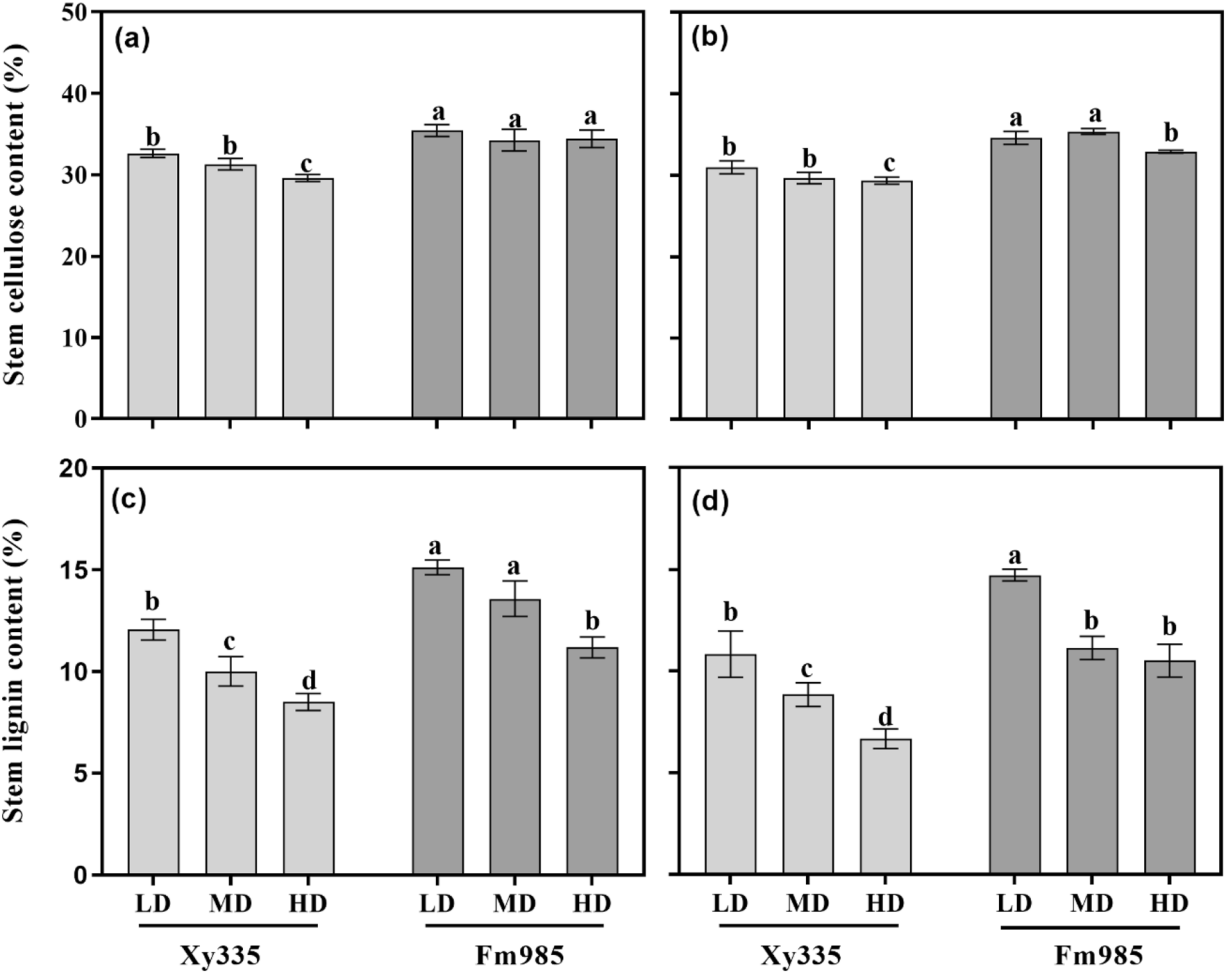
The stem cellulose content in 2018 (**a**) and 2019 (**b**), and lignin content in 2018 (**c**) and 2019 (**d**) under different treatments. LD, MD and HD represent low, medium and high planting densities, respectively. Bars denote the SE of the mean (n = 4).

Stalk bending strength (SBS) was significantly reduced with an increase in PD in both hybrids in two consecutive years (Fig. 3, Table 1). Hybrid Fm985 showed a relatively higher SBS at MD and HD, which were 4.74% and 21.02% higher than Xy335, respectively, in 2018, and 8.24% and 18.19% higher, respectively, in 2019.

Cellulose and lignin are the main structural substances in the maize stems. As shown in Fig. 3, significant differences in stem cellulose content (Fig. 3a, b) and lignin content (Fig. 3c, d) were observed among hybrids and PDs (Table 1). For Xy335, the cellulose and lignin content were 30.89% and 9.49% on average, respectively, which were lower than that of Fm985 (34.57% and 12.71% for cellulose and lignin, respectively). In addition, an increase in PD caused a significant decrease in cellulose content for Xy335, while the differences were not significant for Fm985, except for HD, in 2018. Stem lignin content displayed a significant decrease of 17.01–29.49% for Xy335 and 10.23–26.06% for Fm985, as PD increased from LD to HD in 2018; in 2019, a decrease of 18.25–38.28% and 24.30–28.58%, respectively, were observed.

### Correlation analysis between lodging rate and lodging-related characteristics

**Figure 4:**
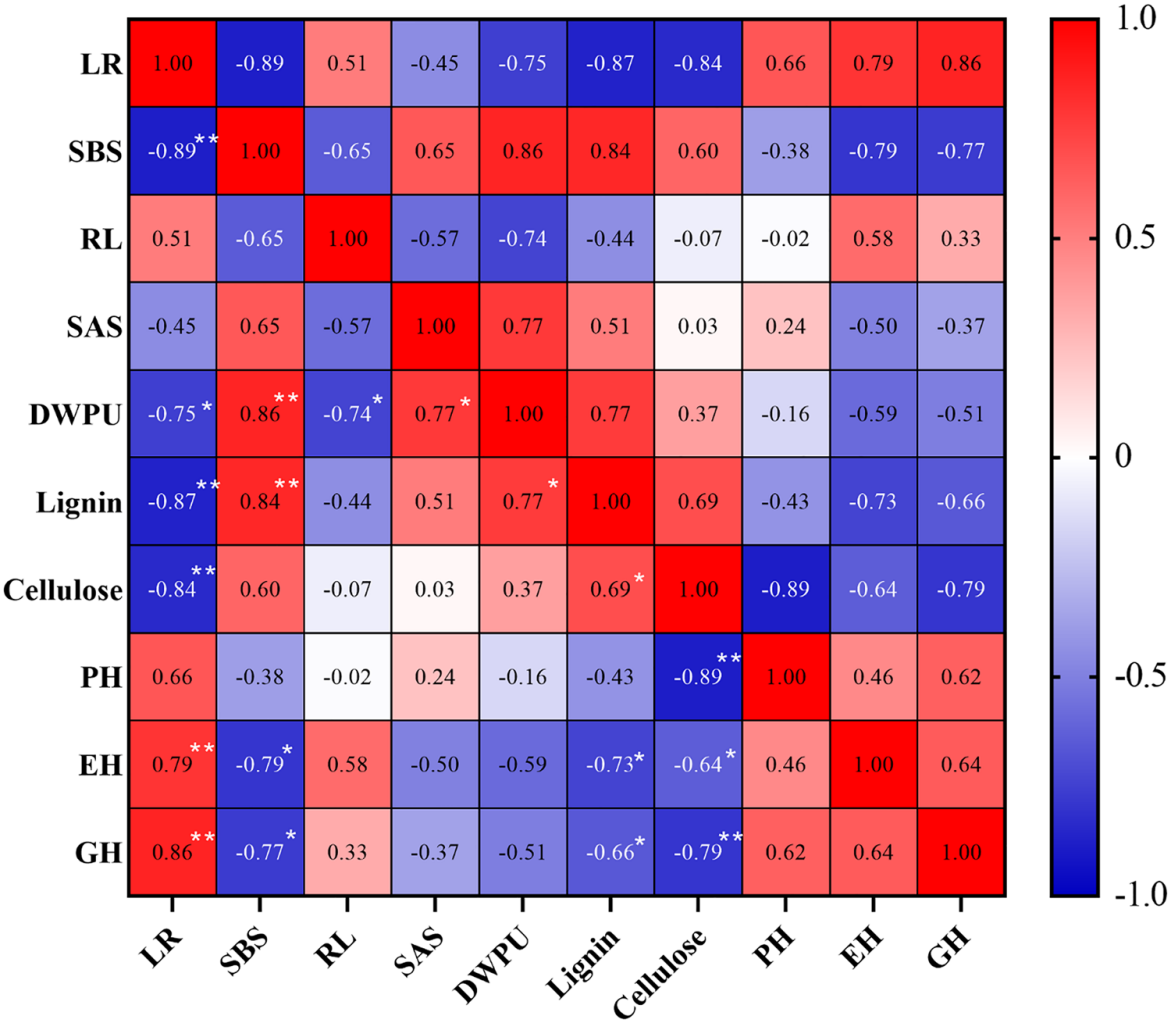
Correlation analysis between lodging rate and lodging-related characteristics. The red and blue colors present a positive and negative correlation, respectively; * and ** indicate differences significant at 0.05 and 0.01 levels, respectively.

Correlation analysis between lodging rate and lodging-related characteristics (Fig. 4) showed that the maize lodging rate was strongly negatively correlated with SBS (r = − 0.89, *p* < 0.01), stem lignin (r = − 0.87, *p* < 0.01) and cellulose (r = − 0.84, *p* < 0.01) content, and DWPU (r = − 0.75, 0.01 < *p* < 0.05) and significantly positively correlated with GH (r = 0.86, *p* < 0.01) and EH (r = − 0.89, *p* < 0.01). Moreover, SBS was highly positively correlated with DWPU (r = 0.86, *p* < 0.01) and stem lignin content (r = 0.84, *p* < 0.01), and negatively correlated with EH (r = − 0.79, 0.01 < *p* < 0.05) and GH (r = − 0.77, 0.01 < *p* < 0.05).

### Path analysis

**Figure 5:**
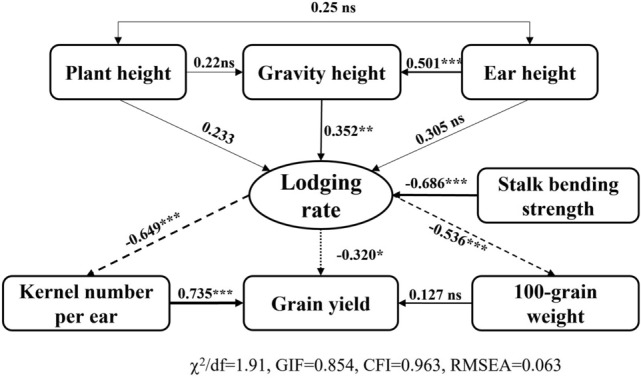
Path analysis of maize grain yield, yield component, SBS and plant architecture characteristics.

**Figure 6:**
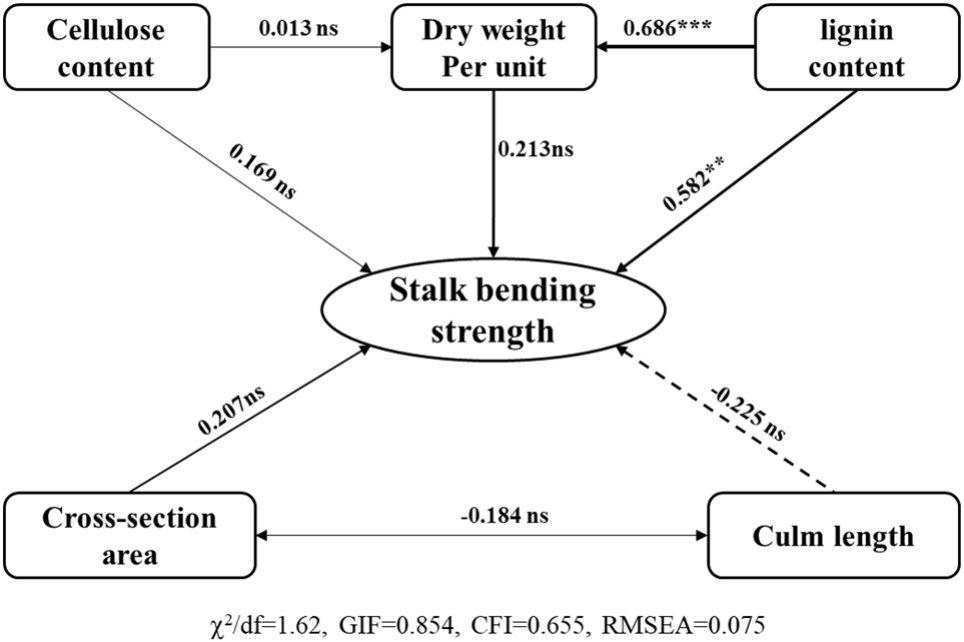
Path analysis of SBS, stem morphology and structural carbohydrates content.

To analyze the causal links between maize grain yield and stem lodging-related morphological and mechanical properties, a structural equation model (SEM) approach was conducted using these characteristics. Kernel number per ear and lodging rate directly contributed to maize grain yield; maize lodging rate (*p* < 0.001), which was directly affected by SBS (*p* < 0.001) and GH, had a negative effect on 100-grain weight and kernel number per ear and an indirect effect on maize grain yield (Fig. 5). In addition, the stem lignin content directly contributed to DWPU and SBS, which were highly related to maize lodging rate and indirectly contributed to maize grain yield (Fig. 5 and 6).

## Discussion

Lodging is a serious problem that hinders grain yield stability and quality in modern crop production^8,21^. Stalk lodging is not only affected by the nature of the cultivar genotypes and agricultural practices^10^ but is also associated with the growth conditions/environment, including wind speed and rainfall^27^. The Super Typhoon Lekima caused heavy rainfall in the mid grain-filling stage in the 2019, resulting in a higher lodging rate and reduction of grain yield, compared with 2018. However, hybrid Fm985 showed a higher grain yield and a lower stalk lodging rate than Xy335 in consecutive years at a high planting density. Therefore, the newly released maize hybrid Fm985 had a higher lodging resistance than the old hybrid Xy335. These results are consistent with the previous studies of Liu et al.^16^ and Luque et al.^24^ who demonstrated that the overall trend in new cultivars is an increase in grain yield and a reduction in stalk lodging in northeast China and other places.

Stalk lodging induces bending and/or breakage of stems during maize growth, generally caused by the damages to vascular bundles^28^, and thereby preventing water transport from root to leaves and impairing phloem transportation of assimilates from the mesophyll into the stalk phloem. Thus, more photoassimilates may be allocated to leaves or stalks than to the ear^2,29^, affecting grain filling and decreasing the kernel weight and effective ear number. Therefore, the decreased yield components of 100-grain weight and ear number were observed in the less lodging-resistant hybrid, Xy335, in the present study.

Maize stalk lodging resistance is also related to many plant characteristics^21^. Increasing planting density brings fiercer competition for solar radiation and the availability of other resources for individual plants, which will be shaped due to plants adaptive regulation^10,30^. Our results are consistent with the previous results of Zhao et al.^28^ who found that the PH and EH were enhanced with the planting density raised from 4.5 × 10^4^ to 8.0 × 10^4^ plant/ha under three N levels. Hybrids with a greater stalk height and ear position are more susceptible to lodging due to higher plant gravity and increased upper fresh weight^21^. In the present study, the maize hybrid Xy335 had a significantly higher PH, EH, and GH at a high planting density, while the differences were non-significant for hybrid Fm985. These results agree with those of previous studies of Cao et al.^8^ and Xue et al.^31^ who also showed that hybrid Xy335 is lodging-susceptible at a high planting density. In addition, plant morphological characteristics have evolutionarily changed with the genetic improvements in the past few decades^16^. A linear positive increase in plant height has been demonstrated with an increase in maize grain yield in the US^32^, as well as in China^1^. However, no differences in grain yield were found between the two hybrids at LD and MD, suggesting that the improved grain yield at HD is associated with enhanced stalking lodging resistance, which contributes to a small yield loss.

Numerous studies have shown that the basal part of the stalk plays an important role in maintaining lodging resistance^21,33^. Stalk morphological characteristics of the basal stem i.e., internodes length, DWPU, and CSA, have a strong correlation with stem wall thickness^17,19^ and therefore influence lodging susceptibility. In the present study, maize hybrid Fm985 had a higher DWPU than Xy335, while no significant differences in CL and CSA were neither found in hybrids nor in planting densities, indicating that DWPU may contribute to the higher bending strength of Fm985 at a high planting density. Previous studies have shown that basal internode DWPU and CSA are correlated with SBS and lodging resistance^8^. The DWPU of the third aboveground internode was the most important factors affecting stem lodging resistance^34^. A recent study confirmed that lodging resistance in maize genotypes had a higher DWPU^35^, which agrees with our results that the newly released maize hybrids Fm985 had a higher DWPU and stem lodging resistance than old hybrid Xy335. A high planting density caused low internode DWPU, which can be attributed to low light intensity by mutual shading^36^, as well as assimilates partitioning between the corn cob and basal stem^35^. In the present study, the newly released maize hybrids Fm985, with erect leaves, may have higher light interception and thus contribute to higher internode DWPU and stalk lodging resistance^27^. However, more possible mechanisms should be addressed.

The maize lodging resistance ability is a complicated genetic mechanism. In addition to the morphological traits of plants, the basal stem chemical composition of cellulose and lignin is also important and contributes significantly to lodging resistance^19^. Culm cellulose can determine stem strength and increase stem rigidity, while lignin, as a vital structural component of the secondary cell walls, can provide strength to plants^37^. Thus, the cellulose and lignin content in the culm plays a critical role in the stalk strength and rigidity of the basal stem, therefore influencing stalk lodging resistance^21^. Significant differences in cellulose and lignin content were observed between planting densities and hybrids. Maize hybrid Fm985 had a higher cellulose and lignin content in the basal stem, thereby providing strong bending strength and stiffness of plant culms. These results agree with Zhang et al.^38^ who demonstrated that high-yield hybrids with strong stem lodging resistance have a higher structural carbohydrate contents in the basal culm of rice. Similar results were shown by Sekhon et al.^39^ in maize. Moreover, transgenic technology has been used to introduce a lignin-deficient mutant gene (*gh2*) to normal plants and has demonstrated that culm lignin content may play a primary role in increasing stalk lodging resistance^40^. Therefore, further studies with detailed analyses will shed more light on the association between structural carbohydrate contents and stalk lodging resistance. Additionally, when the planting density increased from 45,000 (LD) to 85,000 plant/ha (HD), the cellulose and lignin content of Fm985 slightly decreased, while in Xy335, it distinctly decreased, suggesting that developing high lignin varieties may act as an crop breeding strategy to enhance crop stalk lodging resistance.

Stalk lodging occurred mainly depends on two conditions: the strength of external loading conditions (e.g., wind) and its duration^21^; thus, lodging resistance not only depends on basal stem rigidity but is also related to the tensile strength of the plant^41^. Stalk bending strength is a measure used to estimate stalk mechanical strength, both related to stem rigidity and tensile strength^19^. The maize hybrid Fm985 showed a relatively higher SBS at MD and HD in the present study, suggesting that the improved SBS of Fm985 may contribute to its enhanced lodging resistance. Moreover, the correlation between lodging rate and related traits also showed a significant negative effect between SBS and maize stalk lodging rate, indicating that SBS after 35 days of silking could act as an important indicator for estimating lodging resistance in maize breeding or cultivation. SBS is highly associated with stalk morphology and the chemical characteristics of the DWPU, basal internode length, and carbohydrate contents in the culm^28,29^. The correlation analysis also confirmed that SBS is significantly positively related to DWPU (r = 0.86, *p* < 0.01) and stem lignin content ( r = 0.84, *p* < 0.01), and highly negatively correlated with EH (r =  − 0.79, 0.01 < *p* < 0.05), and GH (r = − 0.77, 0.01 < *p* < 0.05). These factors may coordinately play a key role in enhancing the plant stalk lodging resistance and thereby reduce the lodging rate, in contrast to maize grain yield.

Structural equation modeling (SEM) is a powerful, multivariate technique increasingly used in scientific investigations to evaluate multivariate relationships^42^. In recent years, it has been widely used to estimate the direct or indirect effects on plant traits^43,44^. Path analysis using the SEM model showed that kernel number per ear directly contributed to maize grain yield in the present study, and lodging rate was directly affected by SBS. GH had a negative effect on kernel number per ear and 100-grain weight, having an indirect effect on maize grain yield. Stem lignin content, which significantly affected DWPU and SBS, also directly contributed to the grain yield. These results are consistent with the previous studies of Xue et al.^44^ and Shinoto et al.^37^, and agree with our previous study that SBS and DWPU in maize were markedly enhanced by the application of plant growth regulators (PGRs) in Northeast China^8^. In northeast China, maize morphological traits have changed with the improved genotypes over the past few decades. The two typical hybrids were compared in two consecutive years in the present study, and more maize hybrids should be evaluated to verify our results in the future.

## Conclusions

Compared with Xy335, newly released maize hybrid Fm985 had a higher grain yield and a low lodging rate at a high planting density in northeast China. The improved grain yield was significantly correlated with maize kernel number per ear and lodging rate, which were directly affected by SBS and GH, as well as indirectly affected by stem lignin content and DWPU, suggesting that these characteristics which related to the genetic improvement of stalk lodging resistance, play a critical role in grain yield enhancement during modern commercial maize breeding.

## Materials and methods

### Site description

**Figure 7:**
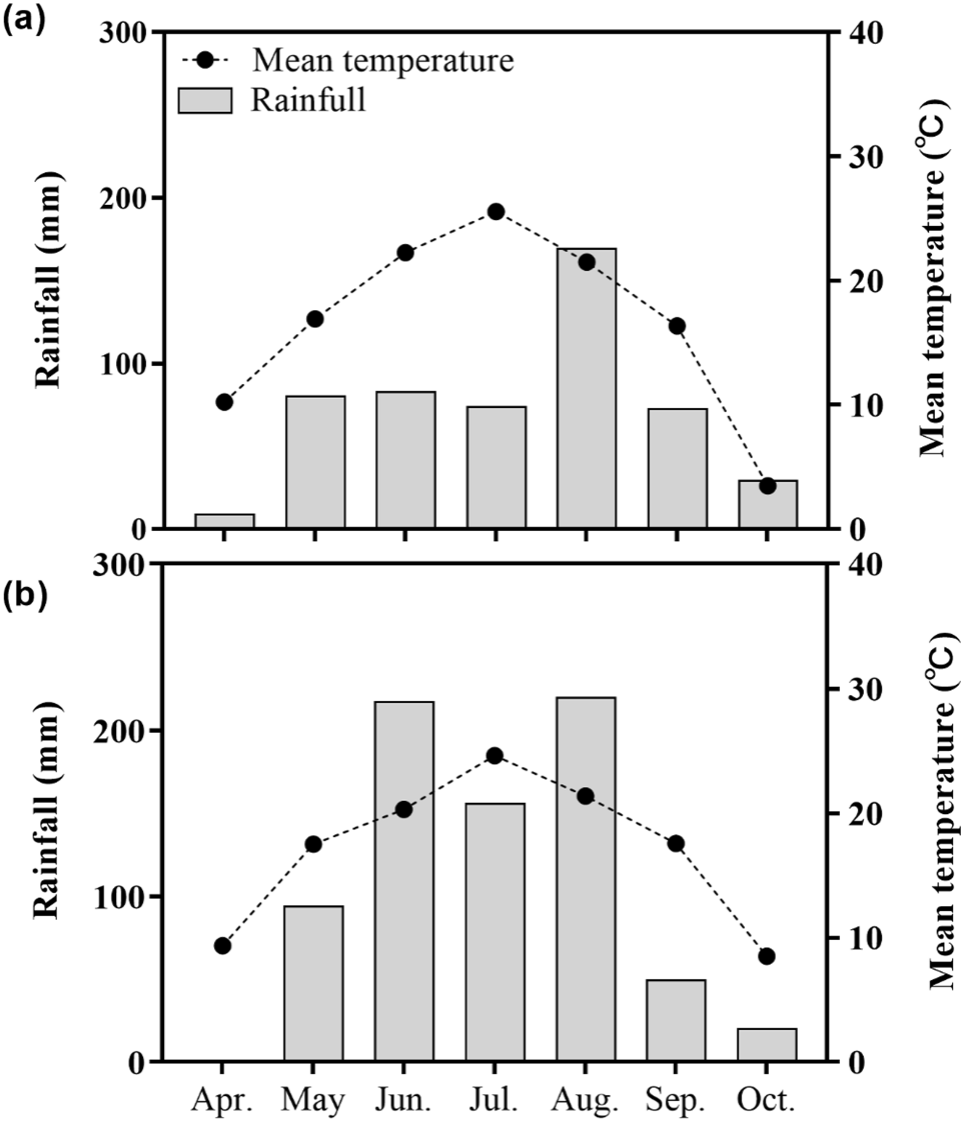
Rainfall and monthly mean temperature recorded during maize growing season (April to October) of experiment in 2018 (**a**) and 2019 (**b**).

The study was conducted at the Jilin Academy of Agriculture Science experiment station (Gongzhuling, Jilin province, China; 43° 31′ 28″ N, 124° 48′ 35″ E) during two consecutive maize-growing seasons in 2018 and 2019. The site is located in the core zone of the “Golden corn belt” in the Songliao plain, northeast of China. This area is a classic rain-fed, long-term spring maize monoculture region with an annual rainfall of about 600 mm. The monthly temperature and precipitation during the time of the experiment were obtained from an automatic meteorological station and are shown in Fig. 7. The experimental field was a typical Black Soil (Mollisols in USDA classification) with a pH of 6.89, containing 22.01 g kg^−1^ organic matter, 1.75 g kg^−1^ total N, 17.60 g kg^−1^ Olsen-P, and 111.1 g kg^−1^ NH_4_OAc-extracted K in the 0–20 cm subsoil layer.

### Experimental setup

Two typical commercial maize hybrids, “Xy335” and “Fm985”, obtained from the Maize Research Institute at the Jilin Academy of Agricultural Science, and Jilin Fumin Seed Co., Ltd., China, respectively, were used in the present study. Both hybrids have large planting areas, with more than one million acres in this zone^43^. Details about the hybrids are presented in Table 4.

**Table 4:** Characteristics of maize hybrids used in the experiments.

Hybrid	Seed company	Year of release	Relative maturity	Density-tolerance	Endosperm type
Xy335	DuPont Pioneer	2004	125	High	Flint
Fm985	Fumin Seed Company	2015	127	High	Flint

The experiment was arranged in a randomized complete block in a split plot arrangement and three replications, with maize hybrids in the main plots and planting density in the subplots. Maize seeds were planted using a manual seeder on May 5 in 2018 and 2019 at three planting densities, namely, 45, 000 (LD), 65, 000 (MD), and 85,000 plant/ha (HD). The sub-plots were 30 m long and 6 m wide (with 10 rows spaced 60 cm apart). To create an equal and sufficient soil growth environment, corn compound fertilizers with 220 kg N ha^−1^, 100 kg P_2_O ha^−1^, and 100 kg K_2_O ha^−1^ were applied at the sowing time according to the recommended soil nutrient management^12^. Weeds in the plots were controlled using herbicides applied before emergence (atrazine and acetochlor) and at the V6 stage (Mesotrione). Pesticides were applied as needed to control insect populations during the maize growth seasons.

### Determination of plant architecture and internode morphological characteristics

**Figure 8:**
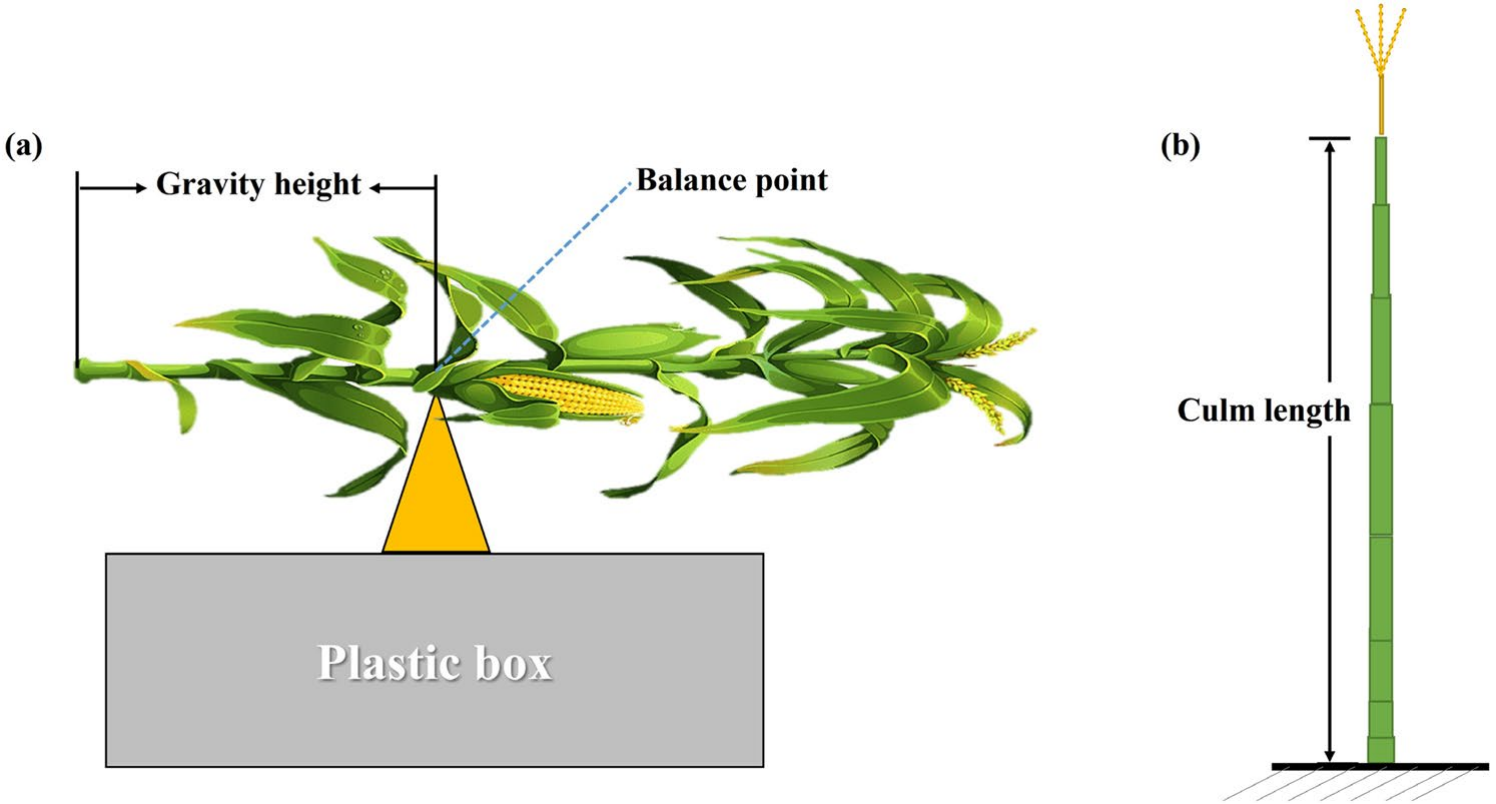
Schematic diagram of the method for measuring the height of the center of gravity (**a**) and culm length (**b**) of maize plants.

At day 35 post silking, five consecutive plants in each plot were randomly selected to measure the plant architecture characteristics of plant height (PH), ear height (EH), and gravity height (GH). PH and EH referred to the distance from the ground to the top of the tassel, and to the node bearing the ear, respectively; GH was measured from the base to the fulcrum of the balance site (Fig. 8a), following the method of Xue et al.^44^.

At physiological maturity, ears were harvested from 10 m^2^ plant samples at the center of each plot. Ten harvested ears were chosen to estimate the ear characteristics, including ear length, kernel number per ear, 100-grain weight, and grain moisture. The maize grain yield and yield components were calculated at a moisture content of 14%.

### Determination of stalk bending strength and internode morphology

**Figure 9:**
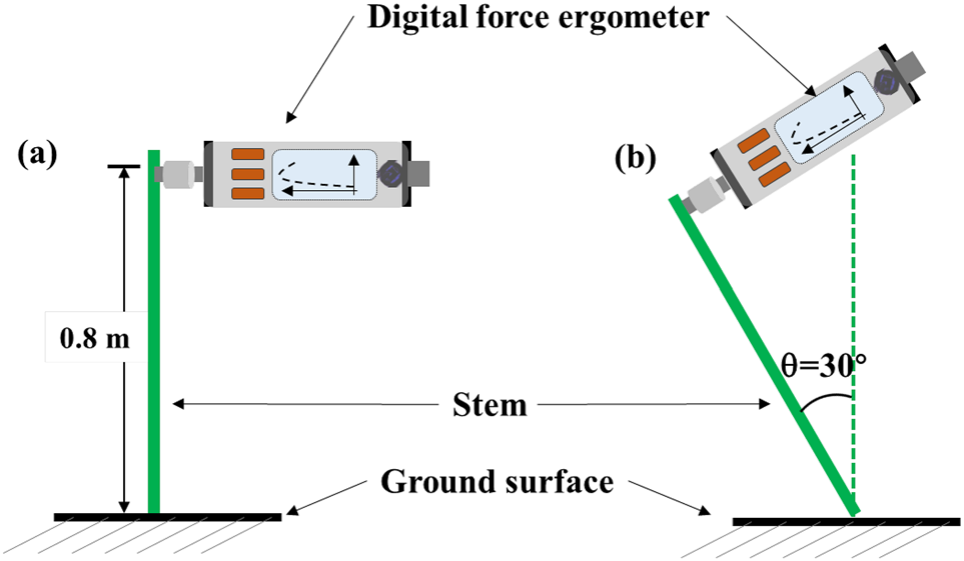
Schematic diagram of the method for measuring the stalk bending strength of maize plants.

To assess maize stalk bending strength (SBS), a self-made instrument using a combination of a digital force ergometer (YYD-2; Zhejiang Top Instrument Co. Ltd) and a circle iron tool^37^ was conducted at day 35 post silking^37,44^. Five adjacent plants in each plot were chosen. After removing the leaves and sheaths of each selected plant, the stems were cut at 0.8 m height from the soil surface, then were pulled until they were 30° from vertical (Fig. 9). The pulling force was determined by the ergometer, and SBS were calculated using the following formula:

$$SBS = \frac{{\sqrt {Stem\;length\;({\text{cm}}) \times Ear\;height\;({\text{cm}})} }}{Horizontal\;pulling\;resistance\;(N)}$$

Meanwhile, the internode morphology of the stem cross section area (CSA) and dry weight per unit length (DWPU) were measured following the method of Cao et al*.*^8^. Culm length (CL) measured using a meter ruler, as shown in Fig. 8b.

### Data analysis

All data were statistically analyzed using SPSS 24.0 (SPSS, Inc., Chicago, IL, USA), including three-way ANOVA, multiple mean comparison using the least significant difference (LSD) test^2^, and Pearson’s correlation coefficients between lodging rate and lodging-related characteristics^45^. A structural equation model (SEM)^43^ was constructed using AMOS software (IBM SPSS AMOS 23.0) to explore the direct and indirect impacts of maize yield components, SBS, plant architecture characteristics, and lodging rate on maize grain yield, as well as the effects of stem morphology and structural carbohydrate content on SBS. This was based on a multivariate approach. Figures were generated in GraphPad Prism 9.0.

### Plant materials legality

We confirm the plants and plant materials used in the present manuscript complied with the laws and regulations of the People’s Republic of China.

## Supplementary Information

Supplementary Information.

## Data Availability

All data are provided in raw data.
