# Supplementary material for: Maize grain yield enhancement in modern hybrids associated with greater stalk lodging resistance at a high planting density: a case study in northeast China
Source: Sci Rep. 2022 Aug 27;12:14647. doi: 10.1038/s41598-022-18908-z (PMC9420147; doi:10.1038/s41598-022-18908-z)
Supplement: Supplementary file 1 — Supplementary Information. [file 41598_2022_18908_MOESM1_ESM.pdf]

Grain yield

		Cultivar_Xy335				Cultivar_Fm985		
2018	*Planting density(×10^4 ha)*	Xy335_1	Xy335_2	Xy335_3		Fm985_1	Fm985_2	Fm985_3
	4.5	8895.65	8570.02	9105.32		9209.79	8993.22	9598.52
	6.5	12123.4	11024.23	11056.7		10122.51	10908.15	12104.26
	8.5	9935.65	10102.38	9815.62		11248.3	10636.46	10125.21
		Cultivar_Xy335				Cultivar_Fm985		
2019	*Planting density(×10^4 ha)*	Xy335_1	Xy335_2	Xy335_3		Fm985_1	Fm985_2	Fm985_3
	4.5	8216.7	8989.4	8991.7		9772.25	9218.25	9038.27
	6.5	11426.3	10121.21	10984.21		10475.46	10128.43	11772.5
	8.5	8922.57	8896.24	9395.41		10766.55	10214.25	9924.32

Ear number per ha

		Cultivar_Xy335				Cultivar_Fm985		
2018	*Planting density(×10^4 ha)*	Xy335_1	Xy335_2	Xy335_3		Fm985_1	Fm985_2	Fm985_3
	4.5	4.5	4.45	4.5		4.5	4.45	4.45
	6.5	6.15	6.21	6		6.41	6.25	6.2
	8.5	8.14	7.89	8.02		8.35	8.12	8.22
		Cultivar_Xy335				Cultivar_Fm985		
2019	*Planting density(×10^4 ha)*	Xy335_1	Xy335_2	Xy335_3		Fm985_1	Fm985_2	Fm985_3
	4.5	4.45	4.5	4.43		4.5	4.35	4.5
	6.5	6.25	6.1	6.25		6.2	6.35	6.22
	8.5	8	7.75	7.88		8.25	8.2	8.14

Kernel amount per ear

		Cultivar_Xy335				Cultivar_Fm985		
2018	Planting density(×10^{4}ha)	Xy335_1	Xy335_2	Xy335_3		Fm985_1	Fm985_2	Fm985_3
	4.5	600.72	541.9	558.48		600.42	571.3	590.22
	6.5	523.74	528	515.1		533.44	515	550.21
	8.5	466.4	477.9	482.16		510.5	480	490.36

		Cultivar_Xy335				Cultivar_Fm985		
2019	Planting density(×10^{4}ha)	Xy335_1	Xy335_2	Xy335_3		Fm985_1	Fm985_2	Fm985_3
	4.5	615.35	540.22	550.34		580.2	541.9	565.48
	6.5	500.24	530.16	533.27		543.74	528.72	520.11
	8.5	480.21	469.23	460.24		476.45	467.25	490.15

100-grain weight

		Cultivar_Xy335				Cultivar_Fm985		
2018	*Planting density(×10^4 ha)*	Xy335_1	Xy335_2	Xy335_3		Fm985_1	Fm985_2	Fm985_3
	4.5	34.48	34.16	35.02		33.69	34.21	32.25
	6.5	32.69	31.83	32.95		33.43	32.54	32.44
	8.5	28.45	28.87	29.28		32.72	30.93	32.95
2019	*Planting density(×10^4 ha)*	Xy335_1	Xy335_2	Xy335_3		Fm985_1	Fm985_2	Fm985_3
	4.5	31.3	31.28	32.2		31.4	31.53	32.46
	6.5	31.28	28.27	29.26		30.99	31.19	30.79
	8.5	28.25	27.47	26.2		29.5	28.11	29.97

Plant height

2018	Planting density ($\times 10^4$ ha)	Cultivar_Xy335					Cultivar_Fm985				
		XY335_1	XY335_2	XY335_3	XY335_4	XY335_5	Fm985_1	Fm985_2	Fm985_3	Fm985_4	Fm985_5
	4.5	320.5	314.8	310.5	328.3	322.75	315.3	300.2	301.3	310	301.4
	6.5	318.5	315.2	326.4	309.43	320.1	290.5	300.5	295.4	295.4	295.35
	8.5	330.5	325.2	323.8	330.21	325.48	295.2	290.3	296.9	300.2	310.25

2019	Planting density ($\times 10^4$ ha)	Cultivar_Xy335					Cultivar_Fm985				
		XY335_1	XY335_2	XY335_3	XY335_4	XY335_5	Fm985_1	Fm985_2	Fm985_3	Fm985_4	Fm985_5
	4.5	315.4	324.8	296.3	320	315.2	308.4	316.5	308.5	302.8	290.4
	6.5	320.5	310.5	290.7	322	320.2	305.2	290.4	296.4	302.3	310.3
	8.5	320.4	320.4	314.3	316.8	330.7	298.4	295.1	290	308.1	288.6

Ear height

2018	Planting density ($\times 10^4$ ha)	Cultivar_Xy335					Cultivar_Fm985				
		XY335_1	XY335_2	XY335_3	XY335_4	XY335_3	Fm985_1	Fm985_2	Fm985_3	Fm985_4	Fm985_5
	4.5	110	108.5	110.6	105.2	105.25	108.25	110	111.5	104.1	105.12
	6.5	106.45	110.5	109	108.1	108.75	112.25	108.37	107.4	109.9	105.24
	8.5	110.45	112.37	115.1	110.5	115.58	115.25	108.33	117.3	105.2	110.24

2019	Planting density ($\times 10^4$ ha)	Cultivar_Xy335					Cultivar_Fm985				
		XY335_1	XY335_2	XY335_3	XY335_4	XY335_5	Fm985_1	Fm985_2	Fm985_3	Fm985_4	Fm985_5
	4.5	108.4	110	107.2	112.1	110.52	105.2	108.2	108.2	102.5	102.5
	6.5	115.4	110.4	113.4	109.4	108.38	110.4	103.3	103.5	109.2	105.37
	8.5	122.1	118.3	134.9	112.4	130.47	110.2	105.5	105.4	111.9	110.24

Gravity height

2018	Planting density ($\times 10^4$ ha)	Cultivar_Xy335					Cultivar_Fm985				
		XY335_1	XY335_2	XY335_3	XY335_4	XY335_5	Fm985_1	Fm985_2	Fm985_3	Fm985_4	Fm985_5
	4.5	105.46	102.28	102.5	106.2	105.24	100	102.25	102.3	104.1	107.35
	6.5	107.22	105.32	108.2	105	107.21	105	103.35	105.2	104.5	106.22
	8.5	115.23	116.42	119.3	114.3	120.24	108.35	100	100.1	106.9	102.22

2019	Planting density ($\times 10^4$ ha)	Cultivar_Xy335					Cultivar_Fm985				
		XY335_1	XY335_2	XY335_3	XY335_4	XY335_5	Fm985_1	Fm985_2	Fm985_3	Fm985_4	Fm985_5
	4.5	108	110	112.1	108.7	113.25	102	115.23	107.4	114.3	115.29
	6.5	113	120	117.5	110	108.39	110	98.35	107.3	103.1	107.24
	8.5	110.35	118	115.7	118.2	122.43	110	102.25	103.6	110.3	108.58

Cross section area (cm^2)

2018	*Planting density (×10^4 ha)*	*Xy335_1*		*Xy335_2*		*Xy335_3*		*Xy335_4*		*Xy335_5*	
		Long axis	short axis	Long axis	short axis	Long axis	short axis	Long axis	short axis	Long axis	short axis
	4.50	27.9	26.5	27.14	26.21	27.3	26.55	27.88	26.84	26.95	25.25
	6.50	25.55	23.48	24.19	22.35	24.5	23.8	25.55	23.77	24.77	22.68
	8.50	25.7	22.84	23.59	25.4	24.81	22.7	23.85	25.57	22.33	19.8

	Planting density(× 10^4 ha)	*Fm985_1*		*Fm985_2*		*Fm985_3*		*Fm985_4*		*Fm985_5*	
2018		*Long axis*	*short axis*	*Long axis*	*short axis*	*Long axis*	*short axis*	*Long axis*	*short axis*	*Long axis*	*short axis*
	4.5	28.97	25.12	26.93	23.65	26.15	22.6	25.26	24.97	26.96	24.95
	6.5	26.68	23.71	25.63	22.74	24.88	21.97	24.9	23.58	24.6	23.75
	8.5	25.19	22.37	24.12	21.54	23.37	20.13	22.85	20.44	24.5	23.2

	Planting density(× 10^4 ha)	*Xy335_1*		*Xy335_2*		*Xy335_3*		*Xy335_4*		*Xy335_5*	
2019		*Long axis*	*short axis*	*Long axis*	*short axis*	*Long axis*	*short axis*	*Long axis*	*short axis*	*Long axis*	*short axis*
	4.5	27.86	26.22	27.66	24.98	27.1	25.72	26.8	24.36	26.18	23.05
	6.5	25.47	23.32	23.72	21.92	27.35	24.2	24.39	21.69	23.34	20.24
	8.5	25.04	21.38	23.42	20.1	24.11	22.45	21.12	18.32	22.6	18.76

2019	*Planting density(× 10^4 ha)*	*Fm985_1*		*Fm985_2*		*Fm985_3*		*Fm985_4*		*Fm985_5*	
		Long axis	*short axis*	*Long axis*	*short axis*	*Long axis*	*short axis*	*Long axis*	*short axis*	*Long axis*	*short axis*
	4.5	26.84	23.88	25.57	22.31	24.81	21.28	23.24	20.15	26.23	26.42
	6.5	25.77	23.61	25.29	21.76	24.6	21.35	24.81	22.7	24.01	22.77
	8.5	24.75	21.56	24.32	22.5	24.3	21.23	22.33	19.85	22.19	24.63

Cellulose content

		Cultivar_Xy335						Cultivar_Fm985				
2018	*Planting density ($\times 10^4$ ha)*	Xy335_1	Xy335_2	Xy335_3	Xy335_4	Xy335_5		Fm985_1	Fm985_2	Fm985_3	Fm985_4	Fm985_5
	4.5	32.8	32.5	32.6	33.79	31.48		36.7	35.42	34.17	33.88	36.98
	6.5	32.1	29.85	31.9	32.72	29.85		35.87	35.24	31.58	34.01	34.45
	8.5	29.65	30.3	28.8	30.74	28.43		34.77	32.4	36.05	33.21	35.6

		Cultivar_Xy335						Cultivar_Fm985				
2019	*Planting density ($\times 10^4$ ha)*	Xy335_1	Xy335_2	Xy335_3	Xy335_4	Xy335_5		Fm985_1	Fm985_2	Fm985_3	Fm985_4	Fm985_5
	4.5	29.5	31.18	32.26	29.36	34.21		34.8	35.84	33.12	38.23	32.63
	6.5	28.54	29.43	31.02	27.43	34.21		36.02	34.88	35.21	34.21	34.25
	8.5	29.35	30.06	28.62	29.85	34.21		33.12	32.57	33.05	33.92	34.9

Lignin content

		Cultivar_Xy335						Cultivar_Fm985				
2018	*Planting density ($\times 10^4$ ha)*	Xy335_1	Xy335_2	Xy335_3	Xy335_4	Xy335_5		Fm985_1	Fm985_2	Fm985_3	Fm985_4	Fm985_5
	4.5	12.22	11.1	12.84	11.88	12.23		15.63	15.31	14.42	14.87	15.37
	6.5	9.28	11.45	9.27	10.3	9.7		13.2	12.29	15.23	14.74	12.41
	8.5	8.81	7.66	9.02	7.06	9.93		10.35	11.08	12.11	12.53	9.83

		Cultivar_Xy335						Cultivar_Fm985				
2019	*Planting density ($\times 10^4$ ha)*	XY335_1	XY335_2	XY335_3	XY335_4	XY335_5		Fm985_1	Fm985_2	Fm985_3	Fm985_4	Fm985_5
	4.5	10.32	9.16	13.02	9.61	12.05		14.62	14.27	15.26	14.42	15.01
	6.5	8.22	8.32	10.02	8.59	9.11		10.11	12.07	11.25	12.3	9.98
	8.5	6.88	5.75	7.42	7.06	6.31		9.35	12.08	10.11	10.03	11
